# Prebiotic effect of inulin-type fructans on faecal microbiota and short-chain fatty acids in type 2 diabetes: a randomised controlled trial

**DOI:** 10.1007/s00394-020-02282-5

**Published:** 2020-05-21

**Authors:** Eline Birkeland, Sedegheh Gharagozlian, Kåre I. Birkeland, Jørgen Valeur, Ingrid Måge, Ida Rud, Anne-Marie Aas

**Affiliations:** 1 Section of Nutrition and Dietetics, Division of Medicine, Department of Clinical Service, Oslo University Hospital, Oslo, Norway; 2 Institute of Clinical Medicine, University of Oslo, Oslo, Norway; 3 Department of Transplantation Medicine, Oslo University Hospital, Oslo, Norway; 4 Department of Gastroenterology, Oslo University Hospital, Oslo, Norway; 5 Unger-Vetlesen Institute, Lovisenberg Diaconal Hospital, Oslo, Norway; 6 Nofima-Norwegian Institute of Food, Fisheries and Aquaculture Research, Ås, Norway

**Keywords:** Prebiotics, Type 2 diabetes, SCFA, Faecal bacteria, 16S rRNA sequencing

## Abstract

**Purpose:**

Compared to a healthy population, the gut microbiota in type 2 diabetes presents with several unfavourable features that may impair glucose regulation. The aim of this study was to evaluate the prebiotic effect of inulin-type fructans on the faecal microbiota and short-chain fatty acids (SCFA) in patients with type 2 diabetes.

**Methods:**

The study was a placebo controlled crossover study, where 25 patients (15 men) aged 41–71 years consumed 16 g of inulin-type fructans (a mixture of oligofructose and inulin) and 16-g placebo (maltodextrin) for 6 weeks in randomised order. A 4-week washout separated the 6 weeks treatments. The faecal microbiota was analysed by high-throughput 16S rRNA amplicon sequencing and SCFA in faeces were analysed using vacuum distillation followed by gas chromatography.

**Results:**

Treatment with inulin-type fructans induced moderate changes in the faecal microbiota composition (1.5%, *p* = 0.045). A bifidogenic effect was most prominent, with highest positive effect on operational taxonomic units (OTUs) of *Bifidobacterium adolescentis*, followed by OTUs of *Bacteroides*. Significantly higher faecal concentrations of total SCFA, acetic acid and propionic acid were detected after prebiotic consumption compared to placebo. The prebiotic fibre had no effects on the concentration of butyric acid or on the overall microbial diversity.

**Conclusion:**

Six weeks supplementation with inulin-type fructans had a significant bifidogenic effect and induced increased concentrations of faecal SCFA, without changing faecal microbial diversity. Our findings suggest a moderate potential of inulin-type fructans to improve gut microbiota composition and to increase microbial fermentation in type 2 diabetes.

**Trial registration:**

The trial is registered at clinicaltrials.gov (NCT02569684).

**Electronic supplementary material:**

The online version of this article (10.1007/s00394-020-02282-5) contains supplementary material, which is available to authorized users.

## Introduction

Advice on diet and physical activity are the cornerstones of treatment of type 2 diabetes for regulation of blood glucose and prevention of long-term complications. Dietary recommendations include a diet rich in dietary fibres [1]. Dietary fibres may have several beneficial effects on glycaemic control, including slowing the rate of nutrient absorption [2, 3], and modifying the gut microbiota. Prebiotic fibres evade degradation in the small intestine and are fermented into short-chain fatty acids (SCFA) in the colon by presumed health promoting gut bacteria, stimulating their growth and activity [4]. The wide-ranging health benefits of bifidobacteria in particular, are well documented [5].

The SCFA produced by gut bacteria, mainly the acetic, propionic and butyric acids, are used as an energy source for the colonocytes and substrates for the hepato-metabolic pathways [6, 7]. The SCFA may also act as signalling molecules by binding to receptors on the enteroendocrine cells, with the potential to increase postprandial secretion of gut hormones and improve regulation of blood glucose [7]. Thus, increased production of SCFA, especially butyric acid, is considered favourable [8–10].

Observational studies have shown that gut microbiota in type 2 diabetes differs from healthy individuals with lower diversity of the microbial community, less of the butyrate-producing bacteria*,* and lower faecal concentrations of SCFA [11–13]. Elevated levels of pathogenic bacteria, and functions related to oxidative stress response, such as enrichment of catalase and increased production of the antioxidant glutathione were also found [12]. Alterations in gut homeostasis such as these are suspected to contribute to the pathophysiology of type 2 diabetes [7].

Improvement of the microbial profile in the gut could benefit individuals with type 2 diabetes in particular, by enhancing the production of SCFA. The inulin-type fructans and galactooligosaccharides are the most studied prebiotic fibres and inulin-type fructans are also extensively used as industrial food ingredients. Numerous trials show that inulin-type fructans supplemented in doses varying between 5 and 30 g per day may increase the abundance of bifidobacteria and SCFA in faeces, and enrich microbial diversity in healthy people and in non-diabetic patients [14–24]. Interestingly, lower levels of bifidobacteria have been reported in individuals with type 2 diabetes compared to healthy individuals, and probiotic supplementation with this genus has been reported to improve glucose tolerance in animal studies [25]. Furthermore, studies conducted in type 2 diabetes patients have also shown that dietary fibres with and without prebiotic abilities could improve glucose metabolism [2, 26]. Yet, to the best of our knowledge, it has not been investigated whether inulin-type fructans have different impact on gut microbiota and fermentation in people with diabetes than in healthy individuals.

The aim of this study was, thus, to evaluate the prebiotic effect of inulin-type fructans on faecal microbiota and SCFA in patients with type 2 diabetes. We hypothesised that treatment with inulin-type fructans for 6 weeks would induce positive changes in the composition of gut microbiota, such as enriching concentrations of bifidobacteria and butyrate producers, increasing the microbial diversity, and increasing concentrations of faecal SCFA.

## Methods

### Trial design

We conducted a randomised, placebo controlled and double-blind crossover trial between February 2016 and December 2017 at the Diabetes Research laboratory, Oslo University Hospital, Aker. Due to high inter-individual variability in the microbial response to dietary interventions, the crossover approach was selected over a parallel design, allowing the participants to serve as their own controls. This study is part of a trial where the primary aim was to investigate the effect of prebiotics on GLP-1 response. These results are not yet published. The trial was approved by the Regional Ethics Committee for Medical and Health Research and registered at clinicaltrials.gov (NCT02569684). Written informed consent was obtained from all patients prior to their inclusion in the study. The study was performed in accordance with the ethical standards laid down in the 1964 Declaration of Helsinki and its later amendments.

### Participants

Adult men and women with type 2 diabetes were invited consecutively as they attended the Diabetes Outpatient Clinic. Participants were also recruited from advertisement in social media, the hospital lobby and pharmacies, and from general practices.

Eligibility for participation was determined at a screening visit at least 4 weeks prior to enrollment. Eligible patients had a BMI ≤ 40 kg/m^2^, HbA_1c_ < 10.0% (86 mmol/mol), and were not treated with insulin or glucagon-like peptide-1 (GLP-1) analogues. Exclusion criteria were fibre intake > 30 g per day, performance of high-intensity exercise, weight changes of > 3 kg within the last month, planned or present pregnancy, drug or alcohol dependence, treatment with antibiotics within the last 3 months, long distance from home to the study centre, and consumption of dietary supplements containing prebiotics or probiotics. At screening, the fibre intake was assessed based on a simplified approach where we asked the potential participants how often they consumed food items known to be important sources of fibre in the Norwegian diet, and their portion sizes. Patients diagnosed with either dementia, organic or functional gastrointestinal diseases, or had cancer within the last 5 years were not included.

In total, 131 patients were assessed for eligibility and 35 were randomised to start with either inulin-type fructans or placebo, of whom 25 completed the intervention (Online Resource 1). Of the ten patients who were randomised, but did not start or complete the intervention, no individuals were excluded or withdrew because of side effects from the supplements or other study-related procedures. One participant was excluded in the faecal microbiota analysis due to one sample with low amounts of extracted DNA.

### Dietary intervention

For two periods of 6 weeks separated by a 4-week washout, the participants consumed 16 g per day of inulin-type fructans (a 50/50 mixture of oligofructose and inulin; Orafti^®^ Synergy1, Beneo GmbH, Germany) and placebo (maltodextrin 16 g per day) in addition to their ordinary diet and in a randomised order. The dose of 16 g was decided after considering the amounts of prebiotics sufficient to induce positive and significant changes in gut microbiota and GLP-1 response against doses low enough to avoid adverse side effects and minimise gastrointestinal discomfort. Trials with healthy adults have demonstrated significant increases in bifidobacteria with doses of inulin-type fructans from 5 g per day [14, 27] and that 10 g per day is preferred rather than 20 g when also taking side effects into consideration [27]. Furthermore, Cani et al. demonstrated that 16-g inulin-type fructans per day induced increased response of GLP-1, and only minor gastrointestinal symptoms in healthy adults [28]. The supplements were powdered, similar in colour and taste, and were wrapped in identical and non-transparent portion packages of 8 g. For adaptation, the participants consumed only 8 g per day during the first week and progressed to 16 g per day for the remaining 5 weeks. The participants added the supplements to food or drinks and consumed it whenever they preferred. They returned unused supplement packages, and the number of unused sachets was used as an estimate of compliance.

### Outcomes and data collection

Before and after the 6-week intervention periods, the participants attended the hospital for visits, where they delivered faecal samples for analysis of microbiota and SCFA. For a comprehensive assessment of diet, the participants filled out food frequency questionnaires (FFQ) before the first intervention period. The participants were instructed to avoid making changes in habitual lifestyle during the trial and to avoid strenuous exercise one day in advance of the visits. They were also told not to make any changes regarding medication during the study and to discontinue diabetes medication two days prior to the visits.

#### Anthropometric measurements

Weight and bioimpedance were measured using a body composition analyser (Tanita BC-418 MA Segmental Body Composition Analyzer) at the four visits, before and after the intervention periods. Height was measured with a standard altimeter. Participants were examined with bare feet wearing light clothing.

#### Assessment of diet

The FFQ is a validated, self-administered, paper-based optical mark readable questionnaire assessing the total diet [29, 30]. Participants were instructed to fill in questionnaires based on eating habits during the last 6 weeks.

#### Faecal collection

The participants were provided with sterile plastic containers to collect faecal samples at home, and instructed to store these instantly in a freezer one day prior to each of the four visits. The samples were brought to the clinic in cooler bags containing freezer blocks and immediately stored at – 80 ℃ for later analysis.

### Microbiota analysis

#### DNA extraction and microbiota analysis

Bacterial DNA was extracted from faecal content (approximately 100 mg) by mechanical and chemical lysis using the DNaeasy PowerSoil HTP 96 Kit (Qiagen), following the manufacture’s protocol. The mechanical lysis step with bead beating was done twice using the FastPrep^®^-96 homogenizer (MP Biomedicals) for 60 s at 1600 rpm. Then, samples were centrifuged for 6 min at 4500 × *g* as described in the protocol. The microbiota was analysed by 16S rRNA amplicon sequencing (2 × 150 bp) of the variable region 4 following an in-house protocol [31], which is presented in detail in supplementary methods of Caporaso et al*.* [32]. The current primers [33–35] have been modified from the original 515F–806R primer pair, with barcodes now on the forward primer and degeneracy added to both the forward and reverse primers to remove known biases. The sequencing was done on a MiSeq (Illumina) at Nofima using pooled polymerase chain reaction (PCR) samples, which were based on triplicate PCRs per DNA sample using sample-specific barcoded forward primers. PhiX Control v3 was included and accounted for 10% of the reads. The MiSeq Control Software (MCS) version used was RTA 1.18.54.

#### Data processing of sequencing data

Data processing of the sequencing reads was performed using the pipelines in Quantitative Insight Into Microbial Ecology (QIIME) v.1.9 [36]. Briefly, the total number of reads was 15,217,265 followed by 9,007,278 reads after joining forward and reverse reads and removal of barcodes that failed to assemble. The sequences were demultiplexed into representative sample taqs and quality filtered, allowing zero barcode errors and a quality score of 30 (Q30), resulting in 7,550,212 sequences. Reads were assigned to their respective bacterial taxonomy (operational taxonomic unit: OTU) by clustering them against the Greengenes reference sequence collection (gg_13_8) using a 97% similarity threshold. Reads that did not hit a sequence in the reference sequence collection were clustered de novo. Chimeric sequences were removed using ChimeraSlayer, and all OTUs that were observed fewer than 2 times were discarded. This resulted in an OTU table containing 15,168 different OTUs, which was based on a total of 6,642,085 read counts. The OTU table was used for microbial (alpha) diversity analysis using equal number of sequences across samples, i.e. alpha rarefaction, where the OTU table was resampled to an even depth of 13,000 sequences per sample. Summary tables at phylum, order, family and genus levels were constructed from the OTU table (i.e. OTU level/species level). The data were transformed by centred log2 ratios, to stabilize the variation and remove dependencies between abundance variables. At any taxonomic level, bacteria groups that were present in less than 50% of the subjects were combined into one group (called “rare”), as it is not possible to make statistical inference on individual rare bacteria groups. Square brackets around taxonomic names (e.g. [Ruminococcus]) are taxa proposed by Greengenes based on genomic trees, but are not verified taxonomies.

### SCFA analysis

Upon analysis, 0.5 g of the faecal material was homogenised after addition of distilled water containing 3 mmol/L of 2-ethylbutyric acid (as internal standard) and 0.5 mmol/L of H_2_SO_4_; 2.5 mL of the homogenate was vacuum distilled, according to the method of Zijlstra et al*.* [37], as modified by Høverstad et al*.* [38]. The distillate was analysed with gas chromatography (Agilent 7890 A, CA, USA), using a capillary column (serial no. USE400345H, Agilent J&W GC columns, CA, USA), and quantified using internal standardisation. Flame ionisation detection was employed. The following SCFA were analysed: acetic, propionic, butyric, isobutyric, valeric, isovaleric, caproic and isocaproic acids. The results were expressed in mmol/kg wet weight. In addition, we calculated the proportional distribution of individual SCFA to total SCFA.

### Gastrointestinal symptoms

After both interventions the participants completed a questionnaire about changes in gastrointestinal symptoms concerning the last 6 weeks (abdominal discomfort, diarrhoea, constipation, bloating, and flatulence) with a word rating scale: much worse, worse, unchanged, better, and much better.

### Sample size

The sample size was calculated based on the expected effects on the primary outcome measurement from the main study, which was change in GLP-1-response to a standardised meal. This estimation was based on results from a drug trial in patients with type 2 diabetes, where changes in GLP-1 response were the primary endpoint [39]. This provided a tentative sample size of 23 patients to achieve 80% power at alpha = 0.05. To account for drop-outs and a possible lower treatment effect due to differences in intervention and design, we added 12 patients, giving a total of 36 patients required for randomisation.

### Randomisation and blinding

Staff not involved in the study performed subject randomisation and product distribution. Randomisation lists were generated using a randomisation command for two by two cross-over studies in Stata 14. All participants and clinical researchers were blinded to treatment allocation and the randomisation key was not broken before all data were collected, the database was washed and the laboratory analyses were performed.

### Statistical analyses

SPSS version 25.0 software was used for descriptive statistics and analyses of biochemical responses. Baseline characteristics are reported as mean (range), (SD) or *n* (%). The variables, total SCFA as well as the individual SCFA, were skewed and their distribution did not improve with log transformation. The effects of inulin-type fructans on SCFA were, thus, analysed using Wilcoxon Signed Rank test and *P* < 0.05 (two tailed) was considered as statistically significant. The results from SCFA analyses are reported as medians (25th–75th percentiles).

The observed variation in microbiota at different taxonomic levels were decomposed by analysis of variance (ANOVA) simultaneous component analysis (ASCA) [40]. The carry-over effect was originally included in the model by the effects *Period* + *Treatment* ×* Week* ×* Period*, but were removed as they were non-significant. The final ASCA model contained a *Subject* effect, accounting for the between subjects variation, and a intervention-specific *Treatment* ×* Week* effect. Post hoc comparisons between factor levels of the intervention design were performed using partial least squares discriminant analysis (PLS-DA) after removing the between-subjects variation [41]. Bacteria that discriminate the prebiotic fibres from placebo and baseline levels were identified by variable importance in prediction (VIP) combined with Pearson correlations between individual bacteria’s group means and class labels [41]. A cutoff of 1.2 was used for VIP and 0.9 for correlation. Effect sizes were calculated as difference between means after prebiotic treatment compared to placebo treatment and baseline values combined.

The microbial diversity, represented by the metrics Observed OTUs, Phylogenetic Distance (PD) whole tree and Chao1, was analysed using a Mixed-Effects Model in Minitab^®^18.1. Treatment, Week and Period were defined as fixed effects and Subject as random.

Partial least squares regression (PLSR) was used to analyse the relationship between microbiota (OTU level) and the different SCFA/metformin users (yes or no) without taking the intervention into account and validated by cross-validation [41]. Variable importance was estimated by the VIP method. Individual variation in effect size of the intervention (subject-specific effect sizes) on the *Bifidobacterium* genus and its OTUs were used to relate against baseline data, i.e. initial level of *Bifidobacterium*, microbial diversity and fibre intake (g/day) characteristics. Data of none identified relationships (i.e. metformin, *Bifidobacterium*, microbial diversity and fibre intake) are not presented. The multivariate statistical analyses were performed using MATLAB (R2018b, The MathWorks Inc.).

## Results

### Patient characteristics

Baseline characteristics of the 25 participants who completed the intervention are presented in Table 1. Forty percent were women, the overall mean age was 63.1 years, BMI 29.1 kg/m^2^, HbA_1C_ 6.9% [52 mmol/mol], and diabetes duration was 4.7 years. Two thirds of participants received glucose lowering medications. The intake of dietary fibre assessed with FFQ at the first visit (baseline) turned out to be higher than expected, as the evaluation of fibre intake at the screening was based on a simpler approach with questioning about how often a few certain food items were consumed and their portion sizes. Apart from a reported higher intake of dietary fibre (mean 32.2 ± 10.3 g/day), the participants characteristics seemed to be representative of patients with type 2 diabetes in Norway.

**Table 1:** Baseline characteristics of study participants

	(*n* = 25)
Women	10 (40.0)
Age (years)	63.1 (41–73)
Fasting glucose (mmol/L)	8.7 (4.0–12.8)
BMI (kg/m^2^)	29.1 (19–39)
HbA1C (%)	6.9 (5.1–9.6)
(mmol/mol)	51.9 (32.2–81.4)
Energy (kcal/day)	2338 (1315–4658)
Proteins (E%)	18.1 (9.6–22.9)
Fat (E%)	36.9 (21.7–44.7)
Carbohydrates (E%)	38.9 (27.4–60.3)
Dietary fibre (g/day)	32.2 (9.6–54.7)
Diabetes duration (years)	4.7 (0.2–20.0)
Diabetes treatment	
Diet	8 (32.0)
Metformin	17 (68.0)
SLGT2 inhibitors^a^	2 (8.0)
DPP-4 inhibitors^a^	5 (20.0)
Sulfonylureas^a^	1 (4.0)
Proton pump inhibitors	0 (0)

Values are mean (range) or *n* (%)

^a^Medication used in addition to Metformin

The compliance was excellent with mean (range) 96.7 (79.2–100.0)% of the prebiotic supplement and 95.7 (77.9–100.0)% of the placebo consumed.

Individual faecal microbiota and effects of inulin-type fructans.

The faecal microbiota was analysed from 24 participants who completed the two crossover periods with four sampling times per individual. Statistical overview of the microbiota data is presented in the online supporting material (Online Resources 2, 4 and 5), also confirming no differences in microbiota composition nor microbial diversity between crossover periods.

**Fig. 1:**
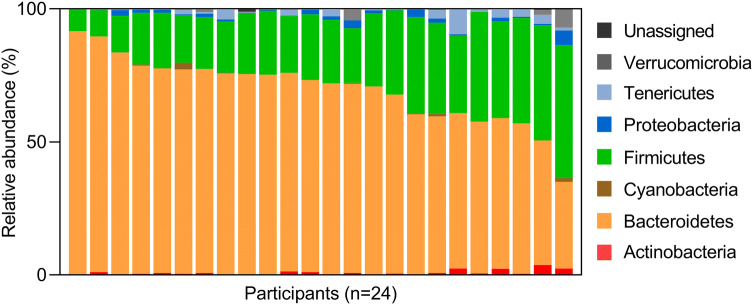
Relative abundance (%) of the dominating phyla in faeces of the participants at baseline

The microbiota data show abundant inter-individual variability of microbiota composition, (explaining > 60% of total variation) and minor effect of the prebiotic fibre (explaining < 2.5%) (Online Resource 2). Overview of the inter-individual variation of phyla at baseline is presented in Fig. 1, showing the gradient distribution of the dominating Bacteroidetes (mean abundance of 69%), with a trade-off with Firmicutes (26%) as the second dominating phylum. Indeed, except for two participants, Bacteriodetes accounted for more than 50% of the microbiota present in the individuals. Tenericutes (1.5%), Proteobacteria (1.2%), Actinobacteria (0.9%), Verrucomicrobiota (0.7%) and Cyanobacteria (0.3%) were also present to a variable degree between individuals.

**Fig. 2:**
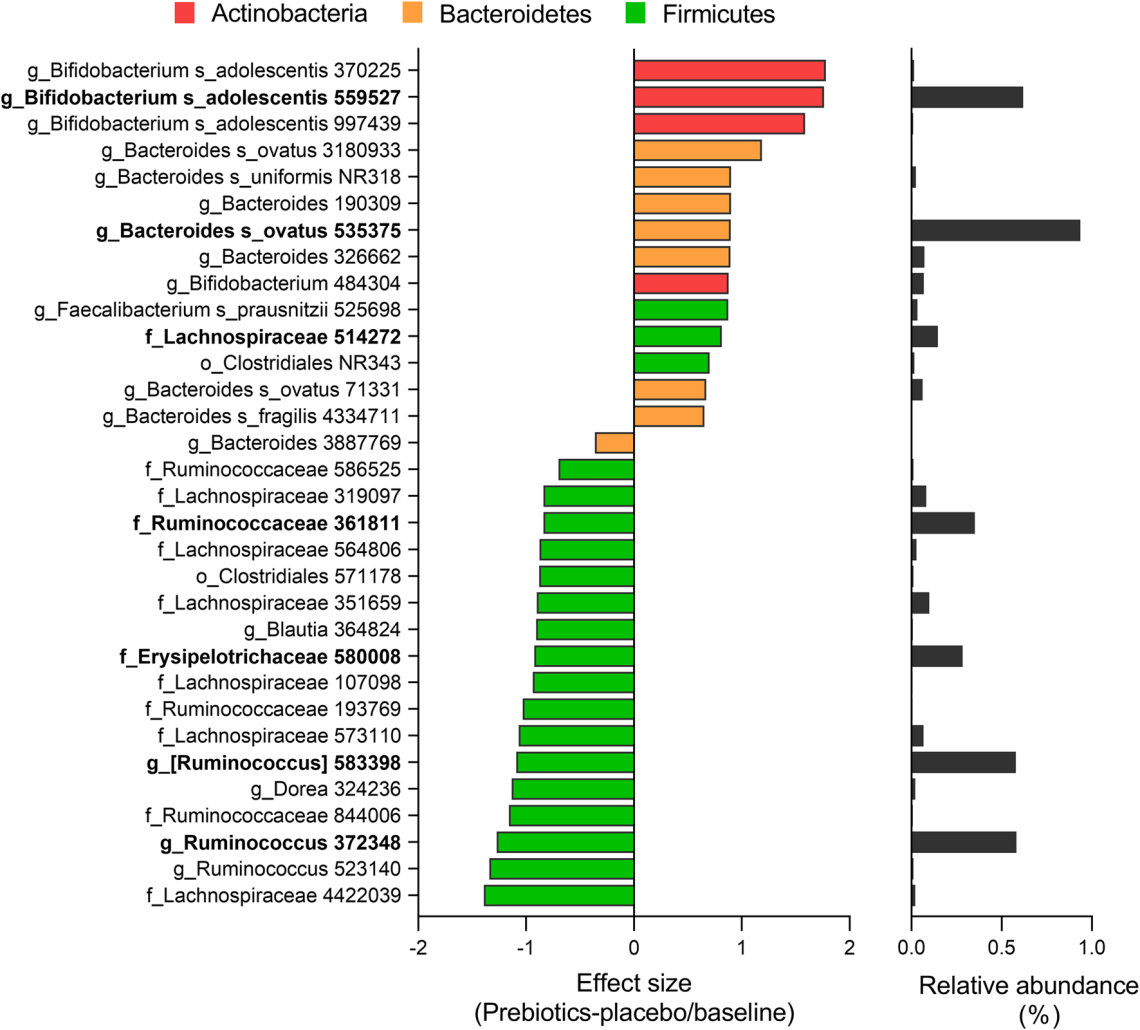
OTUs affected by the prebiotic intervention for 6 weeks sorted by effect size. Effect size is the differences between prebiotic intervention period compared to placebo period /baseline (log2). Dominating OTUs (> 0.1%) are indicated in bold, and the relative average abundance of the OTUs is included at the right. Brackets indicate candidate taxonomy. Bars are coloured according to representative phylum

The moderate changes in microbiota composition after intervention with the prebiotic fibre were explained by only 2.2% and 1.5% of the total variation at phylum and OTU (species) levels, respectively (Online Resource 2). The overall microbiota effect did not reach significance at the phylum level (*p* = 0.091), although Actinobacteria (VIP 1.32) was significantly positively affected by prebiotic fibre compared to placebo and baselines after the 6 weeks of intervention (Online Resource 3). However, the prebiotic fibre had significant effect at the OTU level (*p* = 0.045), with significant impact on 32 OTUs (Online Resource 4). These are presented in Fig. 2, with their representative effect sizes.

Indeed, the three OTUs with highest positive effect sizes were of Actinobacteria and assigned to *Bifidobacterium adolescentis*. *Bifidobacterium adolescenti*s OTU559527 was the most abundant of these OTUs (0.6%). The remaining OTUs positively related to prebiotic fibre intake were not that highly ranked and with less effect sizes, and were mostly of Bacteroidetes origin or Firmicutes. Especially, OTUs within *Bacteroides* were among these, including one dominating OTU assigned to *Bacteroides ovatus*, and three OTUs within Clostridiales, including Lachnospiraceae and *Faecalibacterium prausnitzii*. The OTUs that decreased with the prebiotic fibre were of Firmicutes, including dominating OTUs assigned to the families Ruminococcaceae (*Ruminococcus*) and Lachnospiraceae ([*Ruminococcus*]), all with high effect size. In addition, an OTU of Erysipelotrichaceae declined with the prebiotic fibre.

**Fig. 3:**
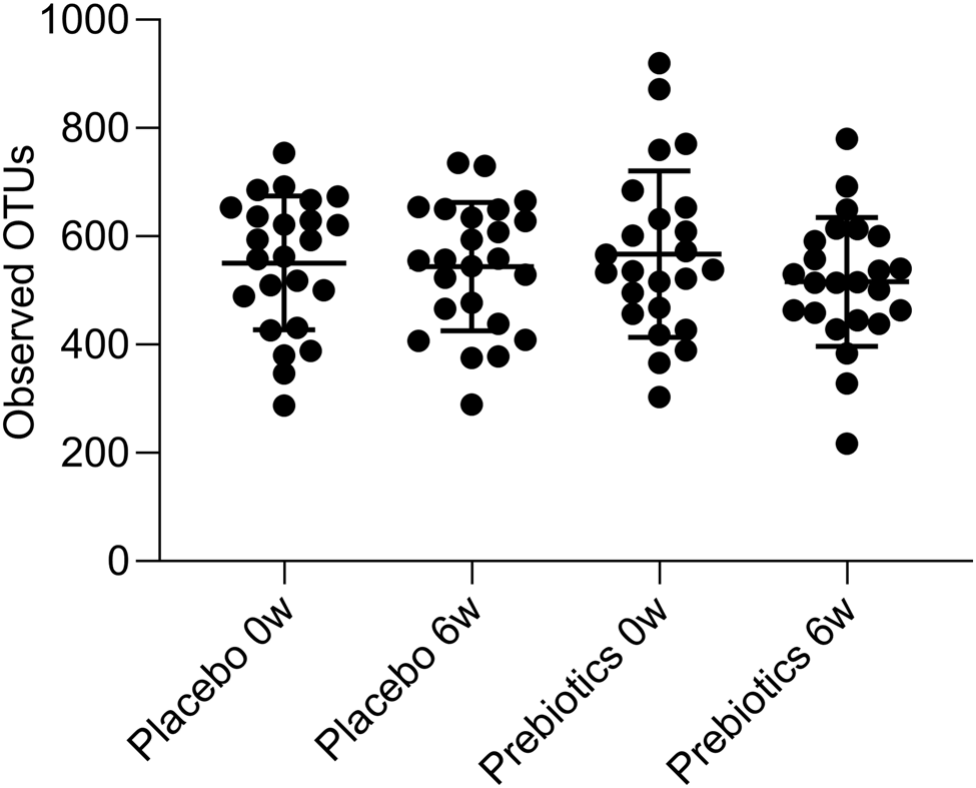
Microbial diversity shown as number of observed OTUs between prebiotics and placebo at baseline (0w) and after treatment period of 6 weeks (6w)

Microbial diversity was not affected by the prebiotic fibre after the 6-week intervention (Online Resource 5), as exemplified with the metrics observed OTUs (Fig. 3).

### Effects of inulin-type fructans on faecal SCFA

The intervention resulted in a significant increase in faecal concentrations of total SCFA (*p* = 0.04), acetic acid (*p* = 0.02), and propionic acid (*p* = 0.04) as compared to placebo (Table 2). There was no difference in effect on butyric acid between the treatments (*p* = 0.19).

**Table 2:** Effects of prebiotics on faecal SCFA in type 2 diabetes

	Prebiotics			Placebo			Between-*p* value
	Baseline	Δ 6w	Within-*p* value	Baseline	Δ 6w	Within-*p* value	
Total SCFA (mmol/kg)	53.95 (43.89–75.23)	8.80 (− 4.7 to 27.44)	0.07	66.53 (58.40–83.50)	− 4.98 (− 24.94 to 12.70)	0.35	**0.04**
Acetic acid (mmol/kg)	31.57 (25.38–40.14)	6.11 (− 3.14 to 13.34)	**0.02**	39.95 (33.95–42.87)	− 4.23 (− 11.94 to 7.45)	0.34	**0.02**
% Acetic acid	56.19 (47.71–60.33)	2.24 (− 6.16 to 9.80)	0.22	53.58 (46.81–62.46)	1.07 (− 3.72 to 6.78)	0.46	0.58
Propionic acid (mmol/kg)	10.02 (6.46–13.33)	1.72 (− 2.49 to 8.57)	0.17	11.75 (8.96–16.25)	− 1.52 (− 3.78 to 1.19)	0.12	**0.04**
% Propionic acid	16.54 (14.81–19.84)	0.06 (− 3.40 to 1.92)	0.96	17.97 (15.26–20.64)	0.26 (− 2.31 to 1.99)	0.90	0.90
Isobutyric acid (mmol/kg)	1.33 (0.87–1.67)	0.05 (− 0.81 to 0.85)	0.99	1.42 (0.91–2.92)	− 0.33 (− 0.61 to 0.21)	0.09	0.70
% Isobutyric acid	2.55 (1.76–3.77)	− 0.05 (− 1.39 to 0.41)	0.19	2.34 (1.38–3.43)	0.03 (− 0.60 to 0.33)	0.49	0.14
Butyric acid (mmol/kg)	9.06 (7.36–15.08)	2.35 (− 1.81 to 4.81)	0.17	10.63 (7.99–16.68)	− 1.18 (− 6.58 to 6.04)	0.53	0.19
% Butyric acid	17.70 (14.20–22.53)	− 0.90 (− 4.33 to 3.86)	0.53	16.30 (14.21–22.31)	− 1.00 (− 5.13 to 4.72)	0.68	0.95
Isovaleric acid (mmol/kg)	2.05 (1.32–2.58)	− 0.01 (− 1.51 to 1.16)	0.76	2.01 (1.37–4.38)	− 0.40 (0.94–0.38)	0.15	0.82
% Isovaleric acid	3.91 (2.61–5.92)	− 0.15 (− 2.29 to 0.86)	0.15	3.52 (1.99–5.32)	0.04 (− 1.01 to 0.77)	0.62	0.09
Valeric acid (mmol/kg)	1.61 (1.06–2.26)	0.06 (− 0.78 to 0.98)	0.80	1.57 (1.19–2.99)	− 0.13 (− 0.50 to 0.28)	0.33	0.44
% Valeric acid	2.93 (2.34–3.42)	− 0.43 (− 1.20 to 0.29)	0.12	2.81 (1.96–3.30)	− 0.02 (− 0.34 to 0.41)	0.80	0.08
Isocaproic acid (mmol/kg)	0.00 (0.00–0.00)	0.00 (0.00–0.00)	0.32	0.00 (0.00–0.00)	0.00 (0.00–0.00)	1.00	0.32
% Isocaproic acid	0.00 (0.00–0.00)	0.00 (0.00–0.00)	0.32	0.00 (0.00–0.00)	0.00 (0.00–0.00)	1.00	0.32
Caproic acid (mmol/kg)	0.09 (0.00–0.62)	0.00 (− 0.14 to 0.05)	0.98	0.15 (0.00–0.15)	0.00 (− 0.17–0.17)	0.98	0.90
% Caproic acid	0.20 (0.00–0.50)	0.00 (− 0.45 to 0.08)	0.33	0.23 (0.00–1.59)	0.00 (− 0.33–0.10)	0.98	0.38

Data are median (25th–75th percentiles). Wilcoxon signed rank test

Significant differences in bold

### Relationship between microbiota and SCFA

**Fig. 4:**
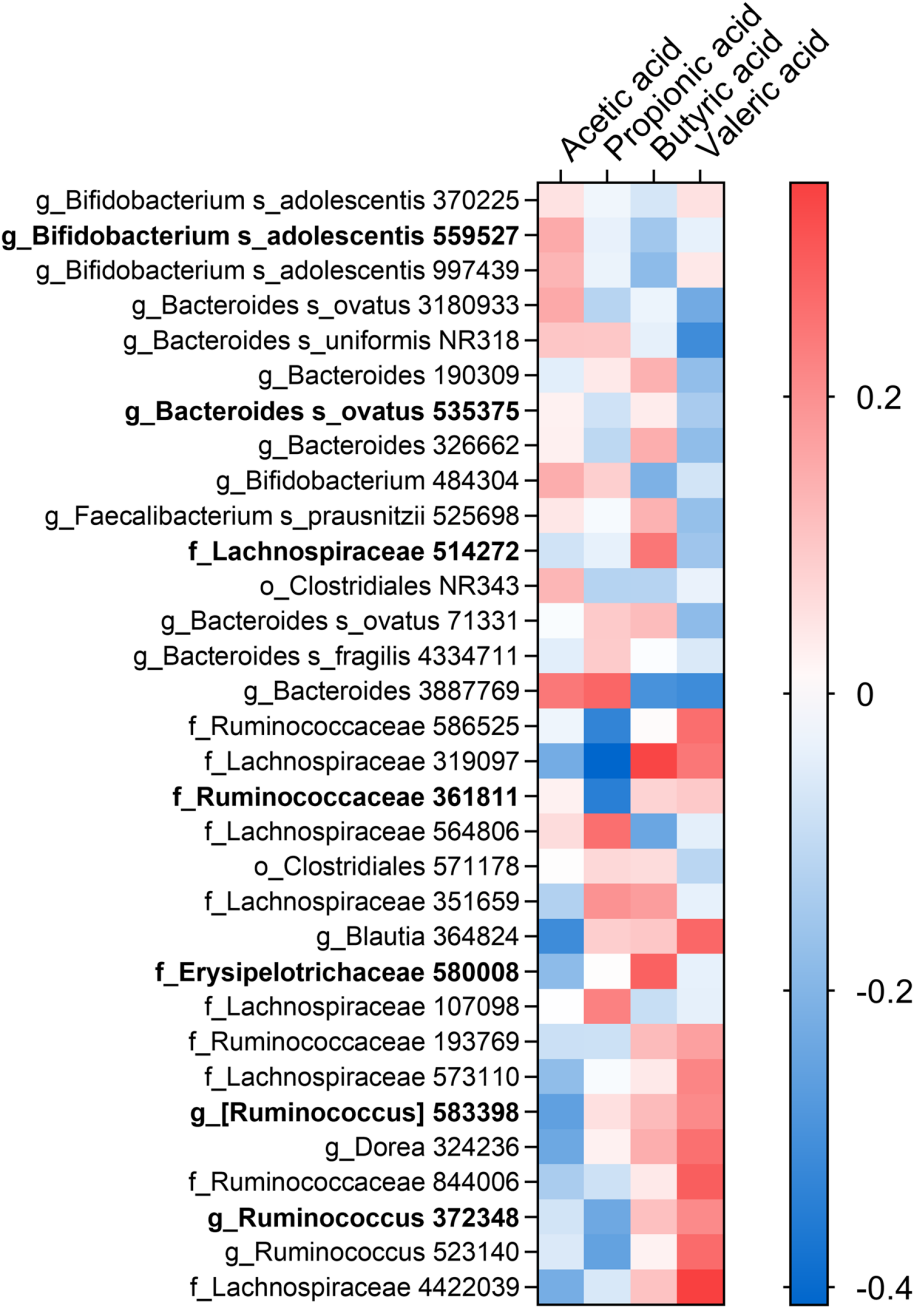
Heatmap of OTUs related to SCFA by PLS regression. Only OTUs affected by the prebiotic intervention are presented and sorted by their effect sizes (as in Fig. 2). Correlation is estimated with Spearman’s rho coefficient, where red is a positive and blue is a negative relation. Asterisk indicates significant relationship (VIP > 1.2). Dominating OTUs (> 0.1%) are indicated in bold

The relationship between microbiota and the SCFA (acetic, propionic, butyric and valeric acid) is presented in a heatmap, only including the OTUs significantly affected by the prebiotic intervention (Fig. 4). A general trend was that acetic acid was positively related to OTUs that increased with the prebiotic fibre. The opposite trend was observed for the OTUs that declined with the prebiotic treatment. Interestingly, the prebiotic affected OTUs of *Bifidobacterium adolescentis* were negatively related towards butyric acid. Only Lachnospiraceae OTU514272 was positively related to butyric acid among the prebiotic affected OTUs. Another trend was that valeric acid was positively related to the OTUs that declined with the prebiotic fibre.

## Discussion

In this randomised controlled trial in patients with type 2 diabetes, we found that 16 g per day of a 50/50 mixture of inulin and oligofructose supplemented for 6 weeks caused an increase in bifidobacteria and SCFA in faeces, compared to maltodextrin. However, the prebiotic fibre had no effect on butyric acid or the overall microbial diversity. To the best of our knowledge, this is the first trial studying the effect of inulin-type fructans on faecal microbiota and SCFA in people with type 2 diabetes.

In planning the present trial, we decided on inulin-type fructans as choice of prebiotic fibres. These are the most studied among prebiotics and a mixture of both long- and short-chain inulin have been proposed to minimise the expected gastrointestinal symptoms [42, 43]. The bifidogenic effect found in the present trial is in accordance with other human studies with doses of inulin-type fructans varying between 5 and 30 g per day, in healthy people and in non-diabetic patients [14–24]. We thus belive a dose of 16 g per day to be sufficient. However, the prebiotic effect on microbiota composition in the present study was moderate, accounting for only a few percentage of variation in the microbiota. This has also been demonstrated in healthy humans given inulin as prebiotic [22], and may be explained by the large individual variation in microbiota between the participants.

Although bifidobacteria are unable to produce butyric acid themselves, they are valuable in cross-feeding where various species metabolise non-digestible carbohydrates through several steps. The bifidobacteria contribute with their ability to degrade fructan chains and, thus, prepare for other species to complete the fermentation [19]. The extensive health benefits of bifidobacteria are well documented [5]. Studies also confirm bifidogenic health benefits of particular interest in type 2 diabetes [44, 45]. Apart from anti-carcinogenic properties and positive effects on blood lipids, trials in humans and mice report that bifidobacteria also may prevent endotoxemia and improve regulation of blood glucose [44–46].

The prebiotic treatment did not have the desired effect of increased microbial diversity in our participants. Tandon et al*.* found increased diversity of faecal bacteria in a healthy population after supplementing fructooligosaccharides (FOS) [47], but others found no or even decreased effect of inulin-type fructans [19, 21, 22]. These studies were all conducted in healthy adults, but with varying treatment doses and degrees of polymerization. The trial performed by Tandon et al. however, stands out with a particular long treatment duration (3 months) and lower treatment dose. This may indicate that it takes longer to affect the microbial diversity than to enhance the abundance of bifidobacteria in the gut when supplementing inulin-type fructans. We chose to limit the duration of the intervention period to 6 weeks to avoid a prebiotic effect of weight loss previously reported [48, 49], as weight loss could potentially have confounded other outcome measures.

Even though the effect of prebiotic fibre on the microbiota composition was moderate, enhanced faecal concentrations of SCFA was detected, indicating changed microbial metabolic activity in the gut. Total SCFA, acetic acid and propionic acid increased significantly. This contrasts the findings in the majority of other clinical trials that measured faecal SCFA after supplementing inulin-type fructans. Only Baxter et al*.* found increased concentrations of total SCFA in faecal samples from healthy individuals supplemented with 20-g inulin per day for 2 weeks, despite no changes in acetic or propionic acid, separately [24]. Others found no or even decreased concentrations of faecal SCFA in healthy adults with normal or overweight after treatment with 5–16-g inulin-type fructans per day for durations between 2 and 12 weeks [18, 19, 21, 23, 24]. Acetic and propionic acid have been linked to mechanisms preserving or improving glucose homeostasis and appear to be anti-carcinogenic, and propionic acid is able to reduce visceral and liver fat [50]. Butyric acid is of particular interest in type 2 diabetes as animal studies report it improves glucose homeostasis by inducing gut production of GLP-1 and peptide YY (PYY) [9] as well as protecting the gut barrier function [51]. However, no significant increase in faecal concentration of butyric acid was detected in the present study. This is in line with the previously mentioned human trials with inulin-type fructans showing no change or even decrease in faecal butyric acid in healthy individuals [19–21]. It is worth noticing that there was a large variability in the measured change in all SCFA, which may be due to individual differences in baseline microbiota, diet and absorption. This can also explain some of the inconsistent findings between studies.

The bifidogenic effect in the present study was related to increase in OTUs assigned to *B. adolescentis*, which were negatively related to butyric acid. Stimulation of *B. adolescentis* is in agreement with other studies using oligofructose and inulin as substrates [24, 52–55]. Fermentability of both the short- and long-chain fructans may have been an advantage of *B. adolescentis*, a capacity shown to be species- and strain dependent among the bifidobacteria [55]. However, bacterial metabolic activity reported in strictly controlled in vitro studies may not occur in the less predictable environment associated with in vivo studies.

Species of *Bacteroides*, e.g. *B. ovatus,* were also enriched by the prebiotic fibre. This genus is known for its genomic capacity to ferment a wide range of polysaccharides into acetic and propionic acid. Capability to ferment both FOS and inulin has previously been shown for *B. ovatus* both genetically and physiologically [56]. It could be speculated that the observed increase in *Bacteroides* species was enhanced by *Bacteroides* being the dominating genus among the type 2 diabetes patients. The butyrate-producing *F. prausnitzii* has in some human studies also been shown to be stimulated by intake of inulin-type fructans [19, 24, 47]. Indeed, *F. prausnitzii* was slightly enriched in this study, as well as an OTU of *Lachnospiraceae* that was also positively related to butyric acid. Still, the increase did not significantly affect the levels of faecal butyric acid. Low levels of butyrate-producing taxas are a well known feature of the type 2 diabetes gut and this may also explain why we did not see significant increase in faecal concentration of butyric acid [11, 12]. Importantly, the faecal concentrations of SCFA is only an estimate of colonic SCFA production. Inulin is rapidly fermented in the proximal colon and most of the SCFA produced are absorbed during transit through the colon, and only few percents remain in the faeces [50]. Apart from the substrate availability, SCFA concentrations in faecal samples are also determined by the absorption rate into the systemic circulation and portal vein, transit time through colon and cross-feeding establishments in the microbiota. Changed faecal SCFA is rather an indication of changed bacterial activity in the gut and thus a valuable measurement when exploring the effect of prebiotic supplements.

Lately, metformin has been shown to affect the gut microbiota, and may, thus, confound the results in clinical trials investigating the composition of gut bacteria in populations with type 2 diabetes [11]. The majority of the participants in our study (68%) used metformin during the intervention, all with a dose that was kept unchanged, and we found no difference in the overall faecal microbiota between participants using metformin or not.

The strengths of this study include the randomised double-blind crossover design, high level of compliance, no dropouts related to the intervention, and assessment of habitual diet and medication known as possible confounders. To minimise the risk of carry-over effects, we included a washout period of 4 weeks. The bacterial response in the gut to dietary intervention occurs within few days and returns to its original state at the same rate when the intervention is discontinued [57]. A remaining effect of prebiotics on faecal SCFA after a 4-week-long washout is, thus, unlikely and no differences between baseline concentrations before and after the washout were found (Online Resource 6).

One clear limitation of this study is measuring of faecal SCFA as a proxy for the colonic production of SCFA. The treatment duration of 6 weeks may also have been too short to enhance the microbial diversity. Another limitation is that the sample size was calculated based on expected effects on the primary outcome measurement from the main study (GLP-1 response) and not on expected effects on composition of the microbiota. However, bifidogenic effect on gut bacteria has been found in comparable studies that have evaluated the effects of inulin-type fructans, both with similar and lower sample sizes [14, 15, 17, 18]. Although we expected some beneficial effects on microbiota composition, diversity and SCFA production, the microbiota analysis should be considered as explorative. Hence, it does not make sense to perform power analysis on selected bacteria groups post hoc. There is also no established method for calculating the power of a multivariate analysis, although some simulation-based approaches have been suggested. However, the fact that moderate changes in total microbiota (1.5%) were observed with relatively low *p* values (< 0.05) indicate that the sample size is sufficiently high.

Results from the FFQ assessment at baseline also showed that our participants slightly exceeded the criteria for allowed fibre intake (mean of 32.2 g per day). This indicates that the study population had higher habitual fibre intake than the general population with type 2 diabetes in Norway, and were on the other hand adherent to the Norwegian dietary recommendations of 25–35-g fibre per day [58]. This may have affected the baseline microbiota composition and diversity and thus its responsiveness to the prebiotic fibre. However, no significant correlation was found between baseline data such as fibre intake, microbial diversity or bifidobacteria levels on the bifidogenic response in the study. This is in contrast to other studies that reported more pronounced bifidogenic response with higher habitual fibre intake [19] and lower baseline levels of bifidobacteria [14, 15, 59, 60]. Nevertheless, regarding the results from the FFQ, we cannot exclude a reporter bias due to the participants’ knowledge of the nature of the study. All dietary assessment methods are known to be biased by both over- and underreporting. This is clearly illustrated by some of the extreme reported intakes of fibre in this study (Table 1). Hence, the data on dietary fibre intake should only be interpreted on group level and not individually.

## Conclusions

In the present study, a daily supplement of inulin-type fructans induced a moderate, but significant increase in faecal levels of bifidobacteria, total SCFA, acetic acid and propionic acid in patients with type 2 diabetes. We were not able to detect any effects on the overall microbial diversity or faecal butyric acid. Our findings imply a moderate potential for these prebiotic fibres to improve the intestinal microenvironment in type 2 diabetes.

## Electronic supplementary material

Below is the link to the electronic supplementary material.

Supplementary file1 (PDF 595 kb)

Supplementary file2 (PDF 12 kb)

Supplementary file3 (PDF 375 kb)

Supplementary file4 (PDF 467 kb)

Supplementary file5 (PDF 179 kb)

Supplementary file6 (PDF 501 kb)

## Data Availability

Data described in the manuscript and analytic code will be made available upon request pending application and approval.

## Abbreviations

ANOVA: Analysis of variance
ASCA: ANOVA simultaneous component analysis
FFQ: Food frequency questionnaire
FOS: Fructooligosaccharides
GLP-1: Glucagon-like peptide-1
MCS: MiSeq Control Software
OUT: Operational taxonomic unit
PCR: Polymerase chain reaction
PLS-DS: Partial least squares discriminant analysis
PLSR: Partial least squares regression
PYY: Peptide YY
Q30: Quality score of 30
QIIME: Quantitative Insight into Microbial Ecology
SCFA: Short-chain fatty acids
VIP: Variable importance in prediction
