# Correction to: Prebiotic effect of inulin‑type fructans on faecal microbiota and short‑chain fatty acids in type 2 diabetes: a randomised controlled trial

**DOI:** 10.1007/s00394-020-02314-0

**Published:** 2020-07-06

**Authors:** Eline Birkeland, Sedegheh Gharagozlian, Kåre I. Birkeland, Jørgen Valeur, Ingrid Måge, Ida Rud, Anne-Marie Aas

**Affiliations:** 1 Section of Nutrition and Dietetics, Division of Medicine, Department of Clinical Service, Oslo University Hospital, Oslo, Norway; 2 Institute of Clinical Medicine, University of Oslo, Oslo, Norway; 3 Department of Transplantation Medicine, Oslo University Hospital, Oslo, Norway; 4 Department of Gastroenterology, Oslo University Hospital, Oslo, Norway; 5 Unger-Vetlesen Institute, Lovisenberg Diaconal Hospital, Oslo, Norway; 6 Nofima-Norwegian Institute of Food, Fisheries and Aquaculture Research, Ås, Norway

## Correction to: European Journal of Nutrition 10.1007/s00394-020-02282-5

The original version of this article unfortunately contained a mistake. The presentation of Fig. 4 was incorrect.

The corrected Fig. 4 is placed in the following page.

**Fig. 4:**
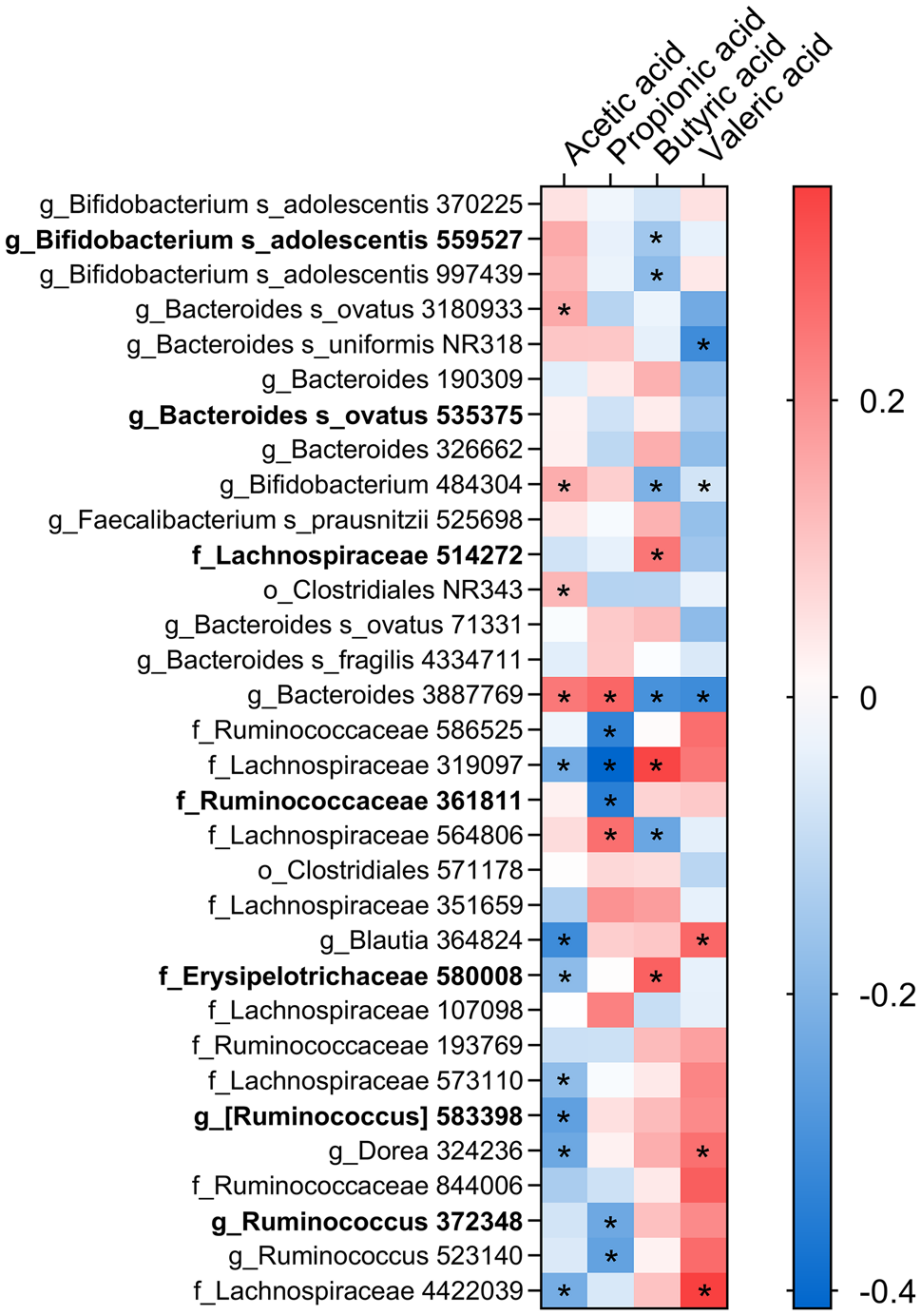
Heatmap of OTUs related to SCFA by PLS regression. Only OTUs affected by the prebiotic intervention are presented and sorted by their effect sizes (as in Fig. 2). Correlation is estimated with Spearman’s rho coefficient, where red is a positive and blue is a negative relation. Asterisk indicates significant relationship (VIP > 1.2). Dominating OTUs (> 0.1%) are indicated in bold

