# Supplementary material for: Prebiotic effect of inulin-type fructans on faecal microbiota and short-chain fatty acids in type 2 diabetes: a randomised controlled trial
Source: Eur J Nutr. 2020 May 21;59(7):3325–38. doi: 10.1007/s00394-020-02282-5 (PMC7501097; doi:10.1007/s00394-020-02282-5)
Supplement: Supplementary file 2 — Supplementary file2 (PDF 12 kb) [file 394_2020_2282_MOESM2_ESM.pdf]

Electronic Supplementary Material

Prebiotic effect of inulin-type fructans on fecal microbiota and short-chain fatty acids in type 2 diabetes: A randomized controlled trial

European Journal of Nutrition

Eline Birkeland[1,2], Sedegheh Gharagozlian[1], Kåre I. Birkeland[2,3], Jørgen Valeur[4,5], Ingrid Måge[6], Ida Rud[6], Anne-Marie Aas[1,2]

Ida Rud and Anne-Marie Aas share last authorship

[1]Section of Nutrition and Dietetics, Department of Clinical Service, Division of Medicine, Oslo University Hospital, Norway, [2]Institute of Clinical Medicine, University of Oslo, Norway [3]Department of Transplantation Medicine, Oslo University Hospital, Norway, [4]Department of Gastroenterology, Oslo University Hospital, Oslo, Norway, [5]Unger-Vetlesen Institute, Lovisenberg Diaconal Hospital, Oslo, Norway, [6]Nofima - Norwegian Institute of Food, Fisheries and Aquaculture Research, Ås, Norway.

Corresponding author: Eline Birkeland, eline.birkeland@ous-hf.no

Online Resource 2. ANOVA table and post-hoc level comparisons of the intervention design at different taxonomic levels.

	Multivariate Analysis of Variance (ASCA)			Post-hoc comparisons between Treatment x Week levels (PLS-DA)	
	Explained variance (%)			Explained variance (%) (cross-validated)	
Taxonomic level	Between subjects effect	Treatment x Week effect	Error	Treatment vs baseline/placebo	Baseline1 vs baseline2
L2	64.9 ($p < 0.001$)	2.2 ($p = 0.091$)	32.9	8.0	0.0
L4	65.7 ($p < 0.001$)	1.7 ($p = 0.195$)	32.5	0.0	0.0
L5	73.9 ($p < 0.001$)	1.1 ($p = 0.457$)	25.0	0.0	0.0
L6	73.2 ($p < 0.001$)	1.3 ($p = 0.123$)	25.5	6.0	0.0
L7	69.9 ($p < 0.001$)	1.5 ($p = 0.049$)	28.6	43.0	0.0
