# Supplementary material for: Prebiotic effect of inulin-type fructans on faecal microbiota and short-chain fatty acids in type 2 diabetes: a randomised controlled trial
Source: Eur J Nutr. 2020 May 21;59(7):3325–38. doi: 10.1007/s00394-020-02282-5 (PMC7501097; doi:10.1007/s00394-020-02282-5)
Supplement: Supplementary file 4 — Supplementary file4 (PDF 467 kb) [file 394_2020_2282_MOESM4_ESM.pdf]

Electronic Supplementary Material

Prebiotic effect of inulin-type fructans on fecal microbiota and short-chain fatty acids in type 2 diabetes: A randomized controlled trial

European Journal of Nutrition

Eline Birkeland[1,2], Sedegheh Gharagozlian[1], Kåre I. Birkeland[2,3], Jørgen Valeur[4,5], Ingrid Måge[6], Ida Rud[6], Anne-Marie Aas[1,2]

Ida Rud and Anne-Marie Aas share last authorship

[1]Section of Nutrition and Dietetics, Department of Clinical Service, Division of Medicine, Oslo University Hospital, Norway, [2]Institute of Clinical Medicine, University of Oslo, Norway [3]Department of Transplantation Medicine, Oslo University Hospital, Norway, [4]Department of Gastroenterology, Oslo University Hospital, Oslo, Norway, [5]Unger-Vetlesen Institute, Lovisenberg Diaconal Hospital, Oslo, Norway , [6]Nofima - Norwegian Institute of Food, Fisheries and Aquaculture Research, Ås, Norway.

Corresponding author: Eline Birkeland, eline.birkeland@ous-hf.no

Online Resource 4. Overview of prebiotic affected OTUs determined by PLS-DA and their correalations to SCFAs (%)

**Bacterial OTUs**			**Prebiotic affected OTUs determined by PLS-DA**			**PLS regression of prebiotic affected OTUs and SCFA (%)**							
						Acetic acid		Propionic acid		Butyric acid		Valeric acid	
						Explained variance: 11%		Explained variance: 27%		Explained variance: 18%		Explained variance: 31%	
OTU	Taxonomy	Average abundance (%)	VIP	Pearson correlation with interaction	Effect size (Prebiotics - placebo/baseline)	Spearmans rho	VIP	Spearmans rho	VIP	Spearmans rho	VIP	Spearmans rho	VIP
370225	p_Actinobacteria; g_Bifidobacterium; s_adolescentis	0.0140	3.05	0.99	1.78	0.05	0.43	-0.02	0.42	-0.07	0.60	0.05	0.56
559527	p_Actinobacteria; g_Bifidobacterium; s_adolescentis	0.6200	3.19	0.99	1.76	0.15	0.92	-0.04	0.23	-0.15	1.28	-0.04	0.61
997439	p_Actinobacteria; g_Bifidobacterium; s_adolescentis	0.0089	2.64	0.99	1.59	0.13	0.98	-0.03	0.27	-0.18	1.33	0.04	0.30
3180933	p_Bacteroidetes; g_Bacteroides; s_ovatus	0.0059	1.79	0.99	1.19	0.15	1.31	-0.12	0.52	-0.03	0.29	-0.23	0.95
NR318*	p_Bacteroidetes; g_Bacteroides; s_uniformis	0.0239	1.57	0.90	0.90	0.10	1.06	0.10	1.05	-0.04	0.65	-0.31	1.50
190309	p_Bacteroidetes; g_Bacteroides; s_	0.0062	1.36	0.99	0.90	-0.05	0.31	0.04	0.45	0.14	0.86	-0.18	0.50

535375	p_Bacteroidetes; g_Bacteroides; s_ovatus	0.9360	1.37	0.92	0.90	0.02	0.04	-0.08	0.60	0.03	0.48	-0.14	0.79
326662	p_Bacteroidetes; g_Bacteroides; s_	0.0728	1.36	0.92	0.90	0.03	0.06	-0.11	1.12	0.14	1.07	-0.18	0.95
484304	p_Actinobacteria; g_Bifidobacterium; s_	0.0695	1.57	0.93	0.88	0.15	1.24	0.08	0.46	-0.21	1.61	-0.07	1.30
525698	p_Firmicutes; g_Faecalibacterium; s_prausnitzii	0.0338	1.35	0.92	0.88	0.04	0.23	-0.02	0.18	0.14	0.96	-0.17	0.50
514272	p_Firmicutes; f_Lachnospiraceae; g_; s_	0.1467	1.48	0.94	0.82	-0.08	0.45	-0.04	0.47	0.24	1.43	-0.16	1.03
NR343*	p_Firmicutes; o_Clostridiales; f_; g_; s_	0.0154	1.26	0.98	0.70	0.13	1.25	-0.12	0.75	-0.12	0.89	-0.04	1.06
71331	p_Bacteroidetes; g_Bacteroides; s_ovatus	0.0624	1.32	0.96	0.67	-0.01	0.05	0.09	0.79	0.12	0.54	-0.18	1.19
4334711	p_Bacteroidetes; g_Bacteroides; s_fragilis	0.0039	1.26	0.94	0.65	-0.05	0.14	0.09	0.81	-0.01	0.18	-0.06	0.41
3887769	p_Bacteroidetes; g_Bacteroides; s_	0.0037	1.26	-0.97	-0.36	0.24	1.78	0.28	1.36	-0.30	2.11	-0.31	1.60
586525	p_Firmicutes; f_Ruminococcaceae; g_; s_	0.0114	1.29	-0.90	-0.70	-0.03	0.30	-0.33	1.81	0.01	0.29	0.26	0.97
319097	p_Firmicutes; f_Lachnospiraceae; g_; s_	0.0818	1.40	-0.99	-0.84	-0.22	1.44	-0.41	2.50	0.33	1.88	0.24	0.98
361811	p_Firmicutes; f_Ruminococcaceae; g_; s_	0.3521	1.29	-0.92	-0.84	0.02	0.21	-0.35	1.86	0.08	0.23	0.10	0.51
564806	p_Firmicutes; f_Lachnospiraceae; g_; s_	0.0272	1.48	-0.95	-0.88	0.06	0.63	0.26	1.45	-0.24	1.96	-0.04	0.78
571178	p_Firmicutes; o_Clostridiales; f_; g_; s_	0.0110	1.32	-0.98	-0.88	0.00	0.01	0.07	0.50	0.06	0.27	-0.11	0.67
351659	p_Firmicutes; f_Lachnospiraceae; g_; s_	0.0987	1.41	-0.96	-0.90	-0.12	1.19	0.20	0.81	0.18	1.05	-0.04	0.59
364824	p_Firmicutes; g_Blautia	0.0077	1.58	-0.99	-0.91	-0.31	2.18	0.09	0.90	0.10	0.90	0.27	1.29
580008	p_Firmicutes; f_Erysipelotrichaceae; g_; s_	0.2837	1.37	-0.93	-0.92	-0.18	1.69	0.00	0.28	0.28	2.06	-0.04	0.70
107098	p_Firmicutes; f_Lachnospiraceae; g_; s_	0.0055	1.42	-0.97	-0.94	0.00	0.40	0.23	1.17	-0.09	0.73	-0.04	0.13
193769	p_Firmicutes; f_Ruminococcaceae; g_; s_	0.0063	1.59	-0.95	-1.03	-0.08	0.67	-0.08	0.74	0.12	0.72	0.17	0.90
573110	p_Firmicutes; f_Lachnospiraceae; g_; s_	0.0671	1.62	-0.90	-1.07	-0.18	1.95	-0.01	0.77	0.04	0.81	0.22	0.88
583398	p_Firmicutes; f_Lachnospiraceae; g_[Ruminococcus]; s_	0.5781	1.88	-0.95	-1.09	-0.26	1.63	0.06	0.38	0.12	0.87	0.21	1.18
324236	p_Firmicutes; f_Lachnospiraceae; g_Dorea; s_	0.0216	1.90	-0.95	-1.13	-0.24	2.03	0.02	0.18	0.14	1.17	0.26	1.44
844006	p_Firmicutes; f_Ruminococcaceae; g_; s_	0.0081	1.80	-0.97	-1.16	-0.14	0.85	-0.08	0.68	0.04	0.22	0.29	1.15
372348	p_Firmicutes; f_Ruminococcaceae; g_Ruminococcus; s_	0.5814	2.07	-0.94	-1.27	-0.07	0.88	-0.24	1.43	0.11	1.02	0.21	1.07
523140	p_Firmicutes; f_Ruminococcaceae; g_Ruminococcus; s_	0.0113	2.06	-0.99	-1.34	-0.06	0.49	-0.25	1.56	0.02	0.34	0.27	1.17
4422039	p_Firmicutes; f_Lachnospiraceae	0.0220	2.08	-0.96	-1.39	-0.22	1.95	-0.06	0.47	0.11	0.88	0.34	1.63

*NR - New.ReferenceOTU
