# Supplementary material for: Prebiotic effect of inulin-type fructans on faecal microbiota and short-chain fatty acids in type 2 diabetes: a randomised controlled trial
Source: Eur J Nutr. 2020 May 21;59(7):3325–38. doi: 10.1007/s00394-020-02282-5 (PMC7501097; doi:10.1007/s00394-020-02282-5)
Supplement: Supplementary file 5 — Supplementary file5 (PDF 179 kb) [file 394_2020_2282_MOESM5_ESM.pdf]

Electronic Supplementary Material

Prebiotic effect of inulin-type fructans on fecal microbiota and short-chain fatty acids in type 2 diabetes: A randomized controlled trial

European Journal of Nutrition

Eline Birkeland[1,2], Sedegheh Gharagozlian[1], Kåre I. Birkeland[2,3], Jørgen Valeur[4,5], Ingrid Måge[6], Ida Rud[6], Anne-Marie Aas[1,2]

Ida Rud and Anne-Marie Aas share last authorship

[1]Section of Nutrition and Dietetics, Department of Clinical Service, Division of Medicine, Oslo University Hospital, Norway, [2]Institute of Clinical Medicine, University of Oslo, Norway [3]Department of Transplantation Medicine, Oslo University Hospital, Norway, [4]Department of Gastroenterology, Oslo University Hospital, Oslo, Norway, [5]Unger-Vetlesen Institute, Lovisenberg Diaconal Hospital, Oslo, Norway, [6]Nofima - Norwegian Institute of Food, Fisheries and Aquaculture Research, Ås, Norway.

Corresponding author: Eline Birkeland, eline.birkeland@ous-hf.no

Online Resource 5. Alpha diversity analysis of the intervention design using Mixed Effects Model (ANOVA).

Alpha diversity metrics	Observed OTUs	ChaoI	PD_whole_tree
Subject	0.004	0.004	0.002
Treatment	0.748	0.886	0.686
Week	0.125	0.146	0.322
Period	0.507	0.427	0.623
Treatment x Week	0.236	0.385	0.282
