# Supplementary material for: Prebiotic effect of inulin-type fructans on faecal microbiota and short-chain fatty acids in type 2 diabetes: a randomised controlled trial
Source: Eur J Nutr. 2020 May 21;59(7):3325–38. doi: 10.1007/s00394-020-02282-5 (PMC7501097; doi:10.1007/s00394-020-02282-5)
Supplement: Supplementary file 6 — Supplementary file6 (PDF 501 kb) [file 394_2020_2282_MOESM6_ESM.pdf]

Electronic Supplementary Material

Prebiotic effect of inulin-type fructans on fecal microbiota and short-chain fatty acids in type 2 diabetes: A randomized controlled trial

European Journal of Nutrition

Eline Birkeland[1,2], Sedegheh Gharagozlian[1], Kåre I. Birkeland[2,3], Jørgen Valeur[4,5], Ingrid Måge[6], Ida Rud[6], Anne-Marie Aas[1,2]

Ida Rud and Anne-Marie Aas share last authorship

[1]Section of Nutrition and Dietetics, Department of Clinical Service, Division of Medicine, Oslo University Hospital, Norway, [2]Institute of Clinical Medicine, University of Oslo, Norway [3]Department of Transplantation Medicine, Oslo University Hospital, Norway, [4]Department of Gastroenterology, Oslo University Hospital, Oslo, Norway, [5]Unger-Vetlesen Institute, Lovisenberg Diaconal Hospital, Oslo, Norway, [6]Nofima - Norwegian Institute of Food, Fisheries and Aquaculture Research, Ås, Norway.

Corresponding author: Eline Birkeland, eline.birkeland@ous-hf.no

Online Resource 6 SCFA baseline concentrations before and after washout[1]

	Baseline concentrations		
	**Before washout**	**After washout**	***p* value**
Total SCFA (mmol/kg)	62.70 (51.91-78.62)	62.67 (49.17-82.95)	0.91
Acetic acid (mmol/kg)	36.00 (30.59-43.23)	36.71 (26.47-41.15)	0.39
Propionic acid (mmol/kg)	11.02 (8.15-15.69)	11.06 (6.89-15.99)	0.98
Butyric acid (mmol/kg)	9.21 (7.29-16.20)	11.95 (8.49-15.03)	0.44

[1]Data are median (25th-75th percentils). Wilcoxon Signed Rank Test. Significant differences in bold
